# Evolutionary conservation of intrinsically unstructured regions in slit-diaphragm proteins

**DOI:** 10.1371/journal.pone.0254917

**Published:** 2021-07-21

**Authors:** Sandeep K. N. Mulukala, Vaishnavi Kambhampati, Abrar H. Qadri, Anil K. Pasupulati

**Affiliations:** Department of Biochemistry, School of Life Sciences, University of Hyderabad, Hyderabad, India; Universita degli Studi di Roma Tor Vergata, ITALY

## Abstract

Vertebrate kidneys contribute to homeostasis by regulating electrolyte, acid-base balance, removing toxic metabolites from blood, and preventing protein loss into the urine. Glomerular podocytes constitute the blood-urine barrier, and podocyte slit-diaphragm (SD), a modified tight junction, contributes to the glomerular permselectivity. Nephrin, KIRREL1, podocin, CD2AP, and TRPC6 are crucial members of the SD that interact with each other and contribute to the SD’s structural and functional integrity. This study analyzed the distribution of these five essential SD proteins across the organisms for which the genome sequence is available. We found a diverse distribution of nephrin and KIRREL1 ranging from nematodes to higher vertebrates, whereas podocin, CD2AP, and TRPC6 are restricted to the vertebrates. Among invertebrates, nephrin and its orthologs consist of more immunoglobulin-3 domains, whereas in the vertebrates, CD80-like C2-set domains are predominant. In the case of KIRREL1 and its orthologs, more Ig domains were observed in invertebrates than vertebrates. Src Homology-3 (SH3) domain of CD2AP and SPFH domain of podocin are highly conserved among vertebrates. TRPC6 and its orthologs had conserved ankyrin repeats, TRP, and ion transport domains, except Chondrichthyes and Echinodermata, which do not possess the ankyrin repeats. Intrinsically unstructured regions (IURs) are conserved across the SD orthologs, suggesting IURs importance in the protein complexes that constitute the slit-diaphragm. For the first time, a study reports the evolutionary insights of vertebrate SD proteins and their invertebrate orthologs.

## Introduction

The vertebrate kidneys regulate the body’s homeostasis by maintaining fluid and acid-base balance and remove toxic metabolic byproducts. Nephron, the vertebrate kidney’s functional unit, consists of the glomerulus and the tubule. The former ensures ultrafiltration of plasma, and the latter participates in selective absorption of the glomerular filtrate [1]. Thus, these two units of nephron work in concert and ensure the final composition of urine. The three anatomical layers that constitute the glomerular filtration apparatus are the glomerular endothelium, glomerular basement membrane, and podocytes. Podocytes are visceral epithelial cells, and they seek greater attention owing to their unique localization and function in glomerular biology. Podocytes are highly specialized cells with distinct morphology and a large nucleus to cytoplasmic ratio. Primary processes of podocytes ultimately branch into regularly spaced foot-processes that enwrap and provide epithelial coverage to the glomerular capillaries. Interdigitating podocyte foot-processes form a modified junction called the slit-diaphragm (SD) [2]. SD serves as size and charge-selective barrier, preventing plasma proteins from filtering into the urine, thus curbing protein loss. Proteins such as nephrin, KIRREL1, podocin, CD2AP, and TRPC6 are essential components of the SD and contribute to podocyte permselectivity.

The SD develops initially as a tight junction during the comma and S-shape stages of glomerular development [3]. Eventually, as the glomerular development progresses, the primary SD structure evolves into a modified tight-adherens junction [4]. Proteins from both tight and adherens junction co-localize at the SD alongside neuronal junction proteins such as nephrin, Kin of IRRE like-1 (KIRREL1) [4]. The SD width ranges from 20-50nm, which is sufficient to curb the passage of proteins from blood into the urinary space [5]. Preliminary evidence suggests that proteins nephrin, KIRREL1, podocin, and Transient receptor potential cation channel-6 (TRPC6) participate in the signaling events that dictate the podocyte morphology [2].

Aberrations in podocyte morphology, including foot-process effacement, lead to proteinuria, hypoalbuminemia, and edema. These are the hallmarks of nephrotic syndrome (NS) [6]. Corticoid therapy is the usual recourse to abate NS. However, patients with steroid-resistant NS (SRNS) do not respond to corticoid therapy. Patients with congenital podocytopathy usually fall into the SRNS category and display mutations in the proteins that constitute the SD. Nephrin, encoded by the *NPHS1* gene, is a critical component of the SD that bridges the distance between interdigitating foot-processes. Mutations in nephrin are in Finnish-type NS patients. Nephrin has a long extracellular domain containing immunoglobulin (Ig)-like modules and a cytoplasmic fibronectin type-3 (FN-3) domain [5]. Similar to nephrin, KIRREL family proteins possess Ig domains. Nephrin and KIRREL1 involve in heteromeric interactions and attribute zipper-like structure to the SD [7]. Podocin is a stomatin family protein and consists of evolutionarily conserved SPFH (stomatin, prohibitin, follitin, and HflC) domain, which is distributed from bacteria to mammals [8]. Podocin, encoded by the *NPHS2 gene*, is frequently mutated and contributes to numerous SRNS cases [9]. CD2-associated protein (CD2AP) interacts with both nephrin and podocin and localizes to the cytoplasmic face of SD [10]. TRPC6, which belongs to the larger family of TRP proteins, is another distinct member of SD [11]. TRPC6 interaction with nephrin and podocin is critical for regulating calcium flux by TRPC6 [12]. Although these five proteins are extensively discussed and form the crux of SD architecture, several other proteins such as P-cadherin and FAT1 also localize to the SD of podocytes [13].

Although invertebrates do not possess typical nephrons, they have many nephron-like components, indicating the vertebrate excretory systems’ complexity is inherited from the invertebrates. For example, the insect nephrocytes and the nephrons in the human kidney share several homologous proteins [14,15]. Interestingly, the SD proteins orthologs in *Drosophila melanogaster* associate as large complexes that closely resemble the SD complex of vertebrates [15]. These observations tingle our interest in investigating the evolution of SD proteins and finding relevant orthologs in different metazoans. Our study focuses on identifying the orthologs of the nephrin, KIRREL1, CD2AP, podocin, and TRPC6 across metazoans. We analyzed the domain composition and intrinsically unstructured regions (IURs) of the identified proteins to assess their evolutionary relationship with the human SD proteins.

## Methods

### Identifying the orthologs

We used five human SD proteins; nephrin (NCBI: NP_004637.1), KIRREL1 (NP_060710.3), CD2AP (NCBI: NP_036252.1), podocin (NCBI: NP_055440.1), and TRPC6 (NCBI: NP_004612.2), to identify the orthologs in metazoan organisms with the complete genome sequences. We used the NCBI’s BLASTp tool against a non-redundant database with a seed value of 6 to initiate the alignment. A threshold value of 0.05 alongside scoring parameters such as BLOSUM62 matrix, gap costs of existence-11, and extension-1 with conditional composition score matrix adjustment was used to perform the reciprocal best hit approach to identify the orthologs in metazoans. In cases where an ortholog of a protein could not be identified, additional searches were performed to identify potential orthologs against the next closest organism. Although we aimed to identify the orthologs for nephrin, KIRREL1, CD2AP, podocin, and TRPC6 across the metazoans, due to limited genome sequence data available for most organisms in various metazoan phyla, our analysis was restricted to only 27 genomes.

### Protein alignment and phylogenetic analysis

We next used the Multiple Sequence Comparison by Log-Expectation (MUSCLE) tool of the MEGA-X software to perform multiple sequence alignment (MSA) of the orthologous proteins FASTA sequences retrieved from the NCBI database. Parameters such as a gap penalty of 2.90, hydrophobicity multiplier of 1.20, neighbor-joining clustering method, and minimum diagonal length (lambda) of 24 were used to perform the MSA. Finally, phylogenetic trees were derived using the individual MSA results for which the maximum-likelihood statistical method (MEGA-X software) alongside other parameters listed in Table 1 were used.

**Table 1:** Parameters and methods used to predict phylogenetic trees for nephrin, KIRREL1, CD2AP, podocin, and TRPC6 proteins and their orthologous sequences.

Parameters	Method
**Statistical method**	Maximum Likelihood
**Test of Phylogeny**	Bootstrap method
**No. of Bootstrap replications**	1000
**Substitution type**	Amino acid
**Model/Method**	Poisson method
**Rates among sites**	Uniform rates
**ML Heuristic method**	Nearest-Neighbor-Interchange (NNI)

### Domain analysis

Domain analysis was performed for SD proteins and identified orthologs. Protein families (Pfam) database catalogs an extensive collection of protein families represented by MSA and Hidden Markov Models. SD proteins and orthologs sequences were submitted in the Pfam database to find matching Pfam families. A default threshold E-value of 1.0 was used for the Pfam HMM search. Pfam version 33.1 was used for identifying domains in the nephrin, CD2AP, podocin, TRPC6 sequences, along with their orthologs. Whereas, for KIRREL1 and its orthologs, Pfam version 34.0 was used. SD proteins participate in protein-protein interactions with the help of intrinsically unstructured regions (IURs) [16]. Therefore, we verified the conservation of IURs among SD proteins and orthologs. We used the DISOPRED 3.1 tool of the PSI-PRED server to extrapolate the IUR’s in the SD proteins and their orthologs. Visual representation of the domains and the IURs in the sequence was done using the illustrator of biological sequences (IBS) software ver. 1.0.

## Results

### SD proteins are confined to vertebrates with few exceptions

**Fig 1:**
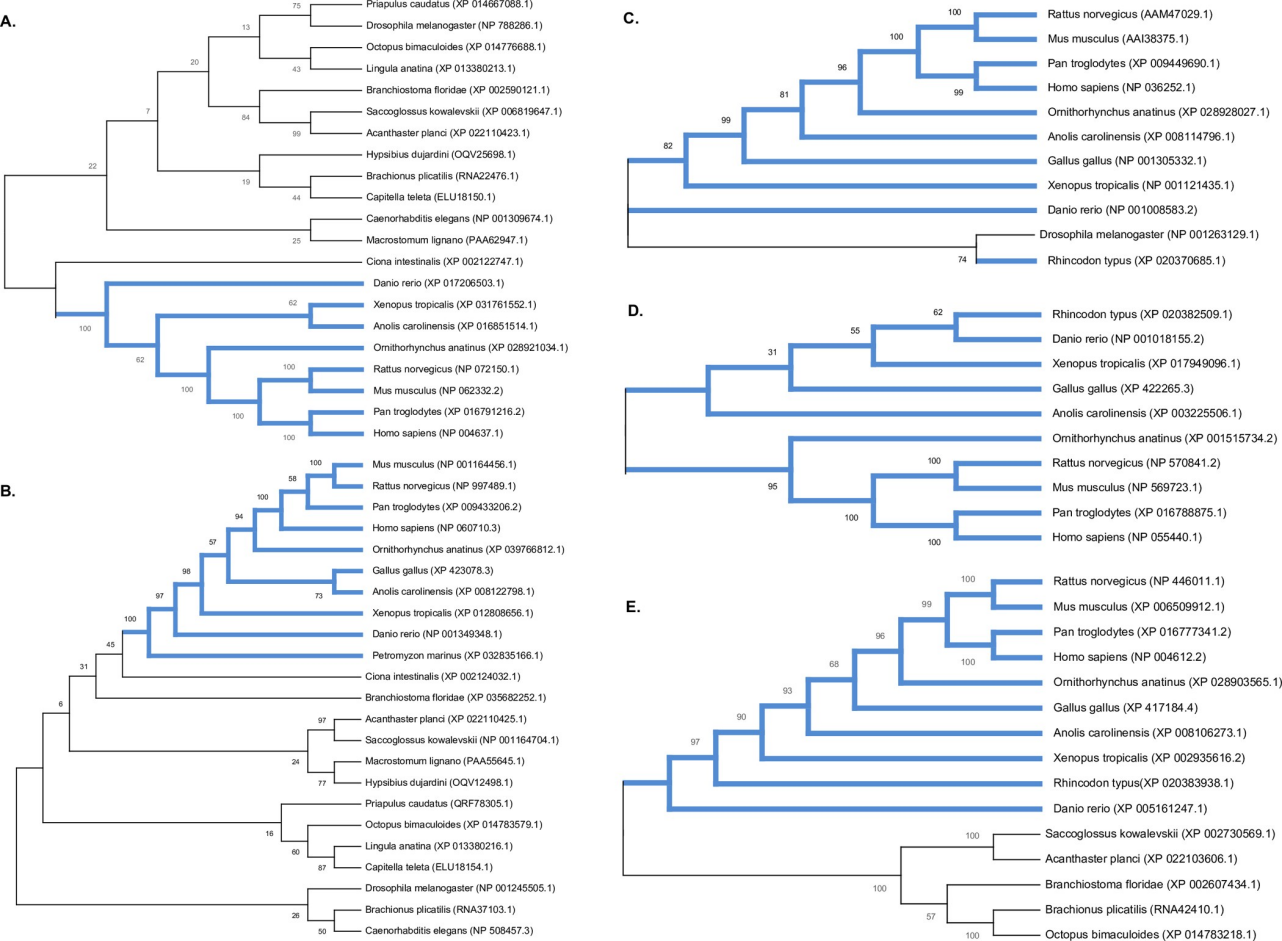
Distribution of SD proteins and their orthologs in vertebrates and invertebrates. A) Nephrin, B) KIRREL1, C) CD2AP, D) Podocin, and E) TRPC6. Only organisms with complete genome sequences were considered for the study. Note: Blue thick lines represent the vertebrate phyla.

We used the reciprocal best hit method to find the orthologs for human SD proteins in both the invertebrate and vertebrates. Table 2 lists the human SD proteins and their orthologs in various invertebrate and vertebrate phyla, along with their respective sequence accession numbers. Our analysis revealed that nephrin and KIRREL1 are present in several organisms ranging from Priapulida to higher vertebrates suggesting a diverse distribution of these two proteins (Fig 1A and 1B). We observed synaptogenesis protein-2 and sticks and stones protein as the orthologs for nephrin in Nematodes and Arthropods, respectively. However, we could not identify the nephrin orthologs in the phyla Onychophora, Nemertea, Phoronida, Cyclostomata, and Chondrichthyes. It is interesting to note that Aves, although they possess the SD structure, we could not identify nephrin or its orthologs. However, we observed nephrin orthologs in the phyla Platyhelminths, Annelida, and Cephalochordata (Fig 1A and Table 2). Our analysis revealed synaptogenesis protein-1 (of Nematoda) and Irregular chiasm C-roughest (of Rotifera and Mollusca) as the orthologs for human KIRREL1. Interestingly, KIRREL3 (of Echinodermata) was identified as the ortholog for human KIRREL1. Similar to nephrin, we were able to identify KIRREL1 orthologs in Platyhelminthes and Annelids, but we couldn’t identify any KIRREL1 orthologs in Onychophora, Nemertea, Phoronida, and Chondrichthyes (Table 2).

**Table 2:** Human SD proteins and their orthologs identified by reciprocal best hit method.

Phylum		Organism	Tax ID	SD proteins and their orthologs names and accession IDs				
				Nephrin	CD2AP	Podocin	TRPC6	KIRREL
**Priapulida**		*Priapulus caudatus*	37621	Nephrin-like (XP_014667088.1)	*-*	*-*	*-*	QRF78305.1
**Nematode**		*Caenorhabditis elegans*	6239	Synaptogenesis protein-2 (NP_001309674.1)	*-*	*-*	*-*	Synaptogenesis protein-1 (NP_508457.3^§^ *(Petromyzon marinus))*
**Tardigrada**		*Hypsibius dujardini*	232323	OQV25698.1	*-*	*-*	*-*	OQV12498.1
**Onychophora**		*Euperipatoides rowelli*	49087	*-*	*-*	*-*	*-*	*-*
**Arthropoda**		*Drosophila melanogaster*	7227	Sticks and stones (NP_788286.1)	CINDR (NP_001263129.1)	*-*	*-*	NP_001245505.1
**Rotifera**		*Brachionus plicatilis*	10195	Nephrin-like (RNA22476.1)	*-*	*-*	RNA42410.1^§^ *(Xenopus tropicalis)*	Irregular chiasm C-roughest (RNA37103.1^§^ (Rattus norvegicus))
**Platyhelminthes**		*Macrostomum lignano*	282301	Hypothetical protein (PAA62947.1)	*-*	*-*	*-*	Hypothetical protein (PAA55645.1)
**Mollusca**		*Octopus bimaculoides*	37653	Nephrin-like (XP_014776688.1)	*-*	*-*	TRPC3 (XP_014783218.1^§^ *(Xenopus tropicalis))*	Irregular chiasm C-roughest-like (XP_014783579.1^§^ *(Petromyzon marinus))*
**Annelida**		*Capitella teleta*	283909	Hypothetical protein (ELU18150.1)	*-*	*-*	*-*	Hypothetical protein (ELU18154.*1)*
**Nemertea**		*Notospermus geniculatus*	416868	*-*	*-*	*-*	*-*	*-*
**Brachiopoda**		*Lingula anatina*	7574	XP_013380213.1	*-*	*-*	*-*	XP_013380216.1
**Phoronida**		*Phoronis australis*	115415	*-*	*-*	*-*	*-*	*-*
**Hemichordate**		*Saccoglossus kowalevskii*	10224	Nephrin-like (XP_006819647.1)	*-*	*-*	TRPC7-like (XP_002730569.1^§^ (*Danio rerio))*	KIRREL-precursor (NP_001164704.1^§^ *(Petromyzon marinus))*
**Echinodermata**		*Acanthaster planci*	133434	Nephrin-like (XP_022110423.1)	*-*	*-*	TRPC3 (XP_022103606.1)	KIRREL3 (XP_022110425.1^§^ *(Petromyzon marinus))*
**Cephalochordata**		*Branchiostoma floridae*	7739	Hypothetical protein (XP_002590121.1)	*-*	*-*	XP_002607434.1^§^ *(Xenopus tropicalis)*	XP_035682252.1
**Urochordata**		*Ciona intestinalis*	7719	XP_002122747.1	*-*	*-*	*-*	XP_002124032.1
**Cyclostomata**		*Petromyzon marinus*	7757	*-*	*-*	*-*	*-*	XP_032835166.1
**Chondrichthyes**		*Rhincodon typus*	259920	*-*	XP_020370685.1	XP_020382509.1	XP_020383938.1	*-*
**Chordata**	**Fishes**	*Danio rerio*	7955	XP_017206503.1	NP_001008583.2	NP_001018155.2	XP_005161247.1	NP_001349348.1
	**Amphibia**	*Xenopus tropicalis*	8364	XP_031761552.1	NP_001121435.1	XP_017949096.1	XP_002935616.2	XP_012808656.1
	**Reptilia**	*Anolis carolinensis*	28377	XP_016851514.1	XP_008114796.1	XP_003225506.1	XP_008106273.1	XP_008122798.1
	**Aves**	*Gallus gallus*	9031	*Absent*	NP_001305332.1	XP_422265.3	XP_417184.4	XP_423078.3
	**Mammalia**	*Ornithorhynehus anatinus*	9258	XP_028921034.1	XP_028928027.1	XP_001515734.2	XP_028903565.1	XP_039766812.1
		*Rattus norvegicus*	10116	NP_072150.1	AAM47029.1	NP_570841.2	NP_446011.1	NP_997489.1
		*Mus musculus*	10090	NP_062332.2	AAI38375.1	NP_569723.1	XP_006509912.1	NP_001164456.1
		*Pan troglodytes*	9598	XP_016791216.2	XP_009449690.1	XP_016788875.1	XP_016777341.2	XP_009433206.2
		*Homo sapiens*	9606	NP_004637.1 (Gene ID: 4868)	NP_036252.1 (Gene ID: 23607)	NP_055440.1 (Gene ID: 7827)	NP_004612.2 (Gene ID: 23607)	NP_060710.3 (Gene ID: 55243)

*Note*: 1. Only the ortholog names of the SD proteins are provided; 2.

’§’ indicates the ortholog of the respective SD protein identified from the organism mentioned in the braces; 3. The cells highlighted in grey denote invertebrate phyla, whereas clear cells indicate the vertebrate phyla; 4. ’-’ indicates the absence of ortholog in those phyla.

Unlike nephrin and KIRREL1, we found CD2AP, podocin, and TRPC6 were restricted mainly to vertebrates with few exceptions. We noticed that only CINDR (CIN85 and CD2AP related) protein in Arthropods showed homology with CD2AP (Fig 1C). Our analysis revealed that podocin was present only in the vertebrates, and we did not find podocin or its related proteins outside the vertebrate phylum (Fig 1D). TRPC3 protein in Mollusca and Echinodermata and TRPC7-like protein in Hemichordates were closely related to TRPC6 (Fig 1E). Further, we identified TRPC6 presence in Rotifers. These results suggest that although nephrin & KIRREL1 distribution is diverse, CD2AP, podocin, and TRPC6 are majorly restricted to vertebrates.

### SD proteins and their orthologs share conserved domains

**Fig 2:**
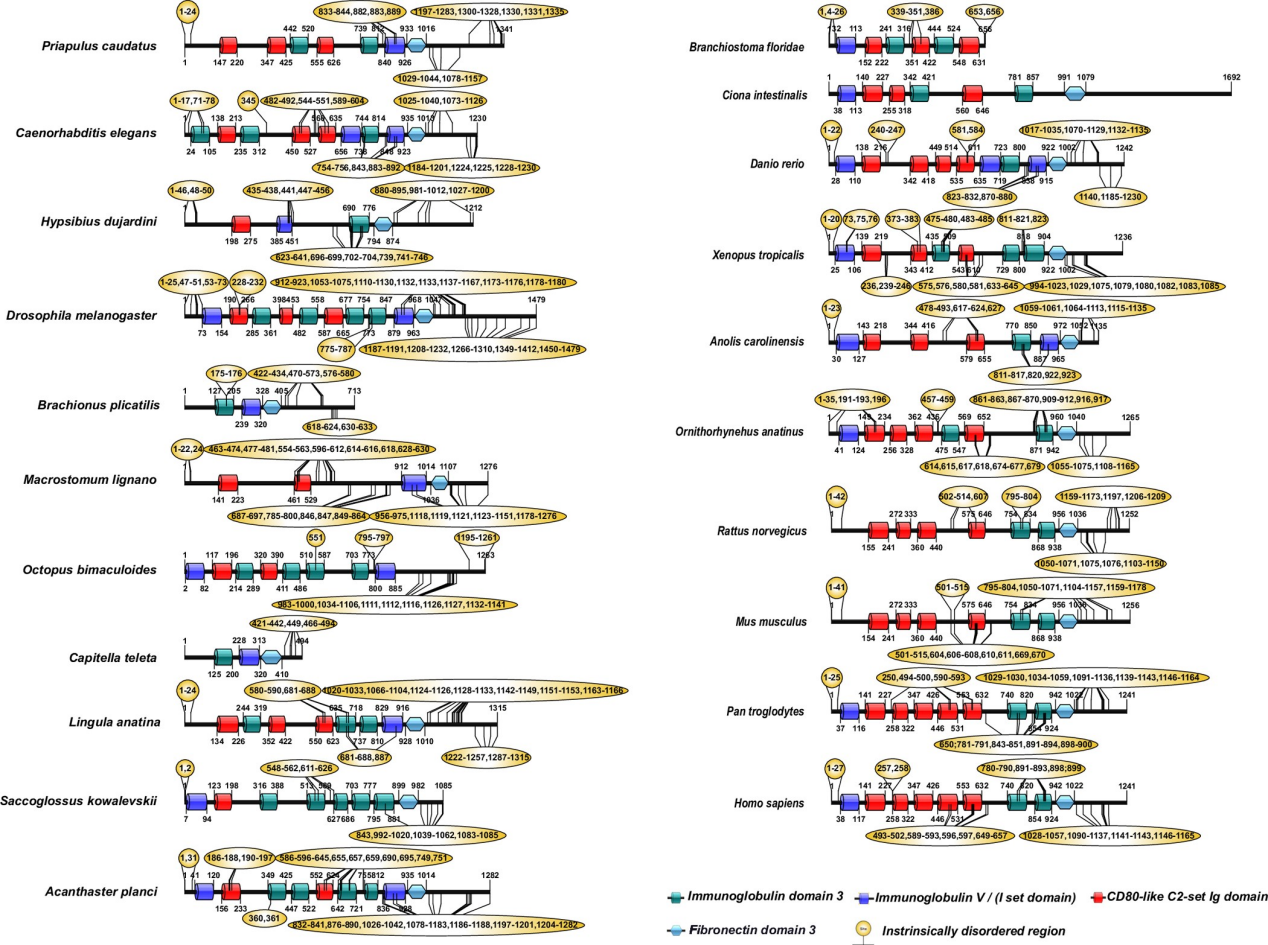
Domain organization in nephrin and its orthologs. Different colors represent Ig domain subsets: Ig3 as green, CD80-like C2-set as red, IgV as blue, FN-3 as light blue, and intrinsically disordered regions as yellow bubbles.

**Fig 3:**
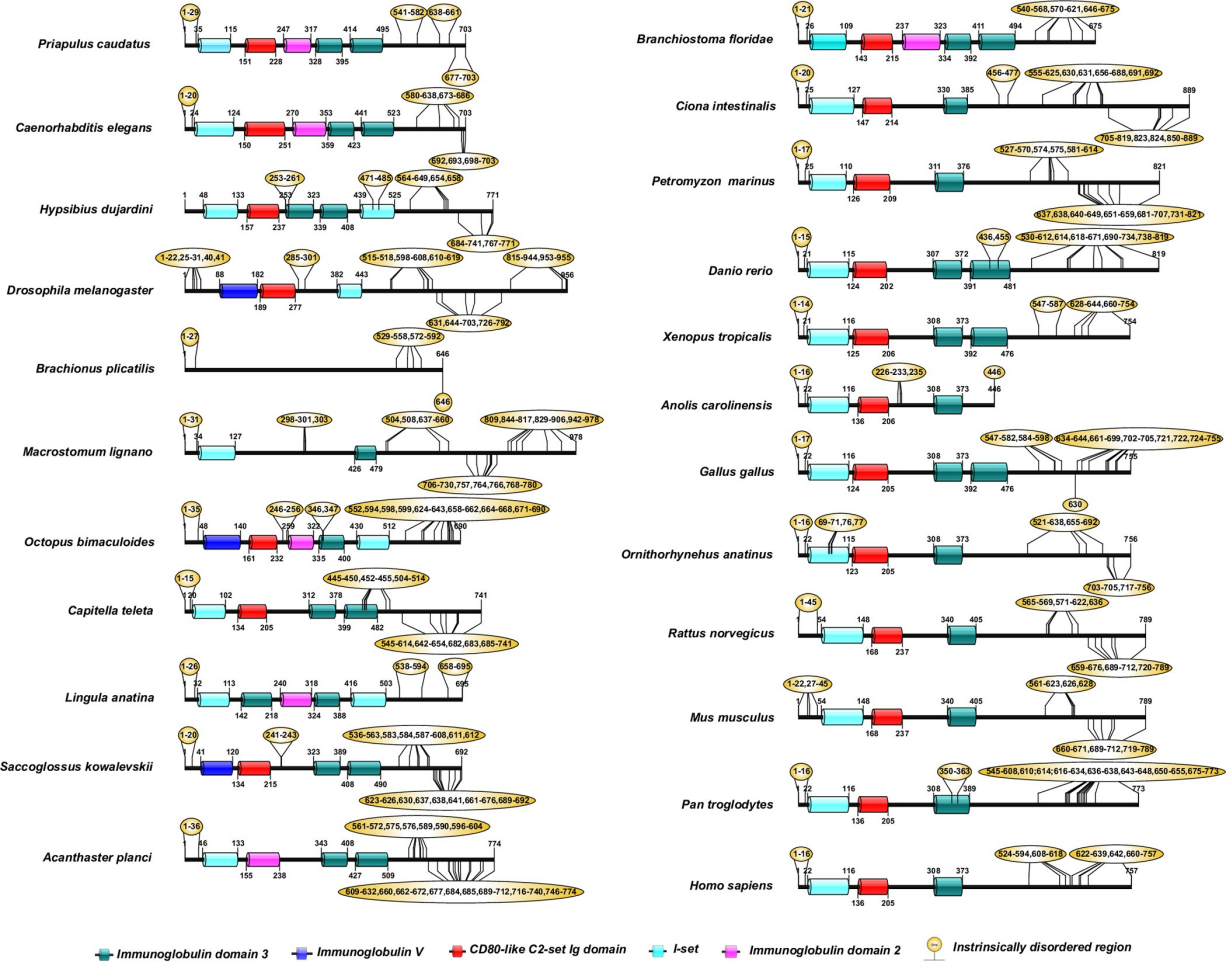
Domain organization in KIRREL1 and its orthologs. Different colors represent the Ig domain subsets: Ig2 as pink, Ig3 as green, I-set as light blue, CD80-like C2-set as red, Ig V-set as dark blue, and intrinsically disordered regions as yellow bubbles.

As we observed the distribution of SD proteins and their orthologs in various phyla, predominantly in the vertebrates, we next assessed the evolutionary accumulation and conservation of unique domains in these proteins. Human nephrin Ig domains consist of one Ig5, two Ig3, five CD80-like C2-set domains, and a fibronectin type-3 (FN-3) domain (Fig 2) [17]. Invertebrates nephrin and its orthologs had one to five Ig3 domains compared to vertebrates with about one or two Ig3 domains. In contrast, nephrin in vertebrates had three to four CD80-like C2-set domains than invertebrates with about one to three domains. For example, Arthropoda’s sticks and stones protein comprises two-Ig5, four-Ig3, and three-CD80-like C2-set domains, whereas nephrin in Mammals consists of two-Ig3, one-Ig5, and four-CD80-like C2-set domains (Fig 2). Furthermore, in Mollusca and Cephalochordate, nephrin is devoid of the FN-3 domain (Fig 2). Unlike an earlier study that reported five Ig domains in human KIRREL1 [18] our analysis predicted only three Ig domains (Ig3, I-set, and CD80-like C2-set) in humans and other vertebrates except in Aves, which had an extra Ig3 domain (Fig 3). In invertebrates, KIRREL1 and its orthologs possess >3 and ≤5 Ig domains except in Platyhelminthes, which had one Ig3 and I-set domains, and Arthropods had one of each I-set, Ig V-set, and CD80-like C2set domains (Fig 3). It is noteworthy that Cephalochordata, Echinodermata, Brachiopoda, Mollusca, Nematoda, and Priapulida displayed Ig2 domain variant and Echinodermata, Annelida, and Arthropoda had Ig V-set domain variant (Fig 3). Interestingly, our analysis method was unable to predict any domains in Rotifers.

**Fig 4:**
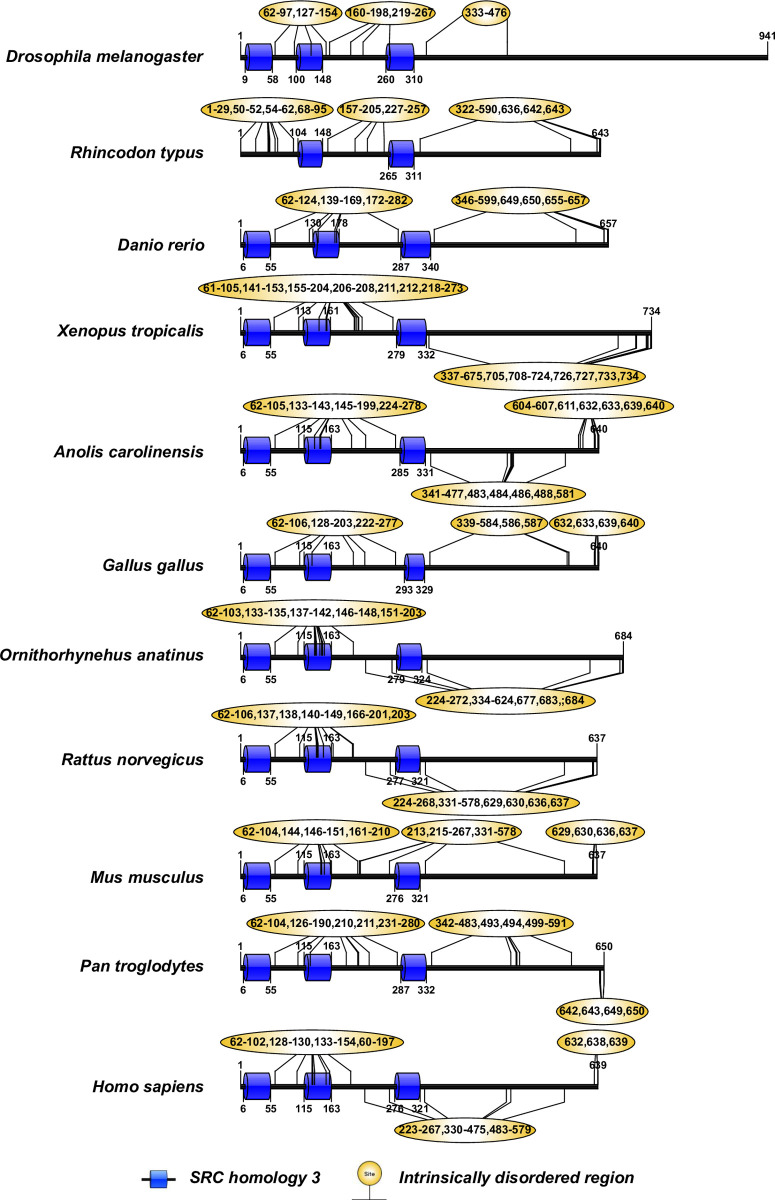
Domain organization in CD2AP and CINDR sequences. Note: Yellow bubbles represent the intrinsically disordered regions.

**Fig 5:**
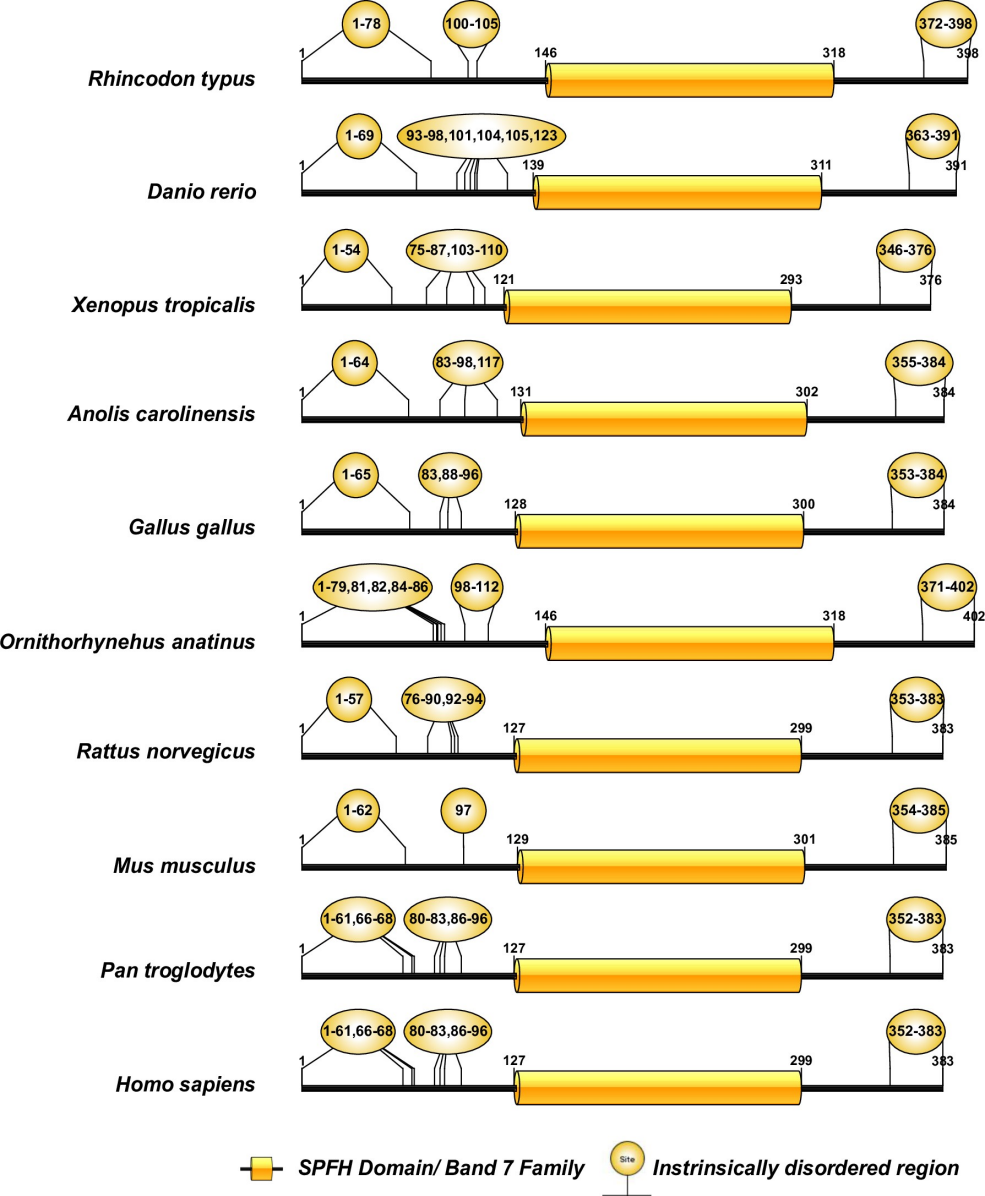
Domain organization of podocin sequences of the chordate phylum. Note: Yellow bubbles represent the intrinsically disordered regions.

**Fig 6:**
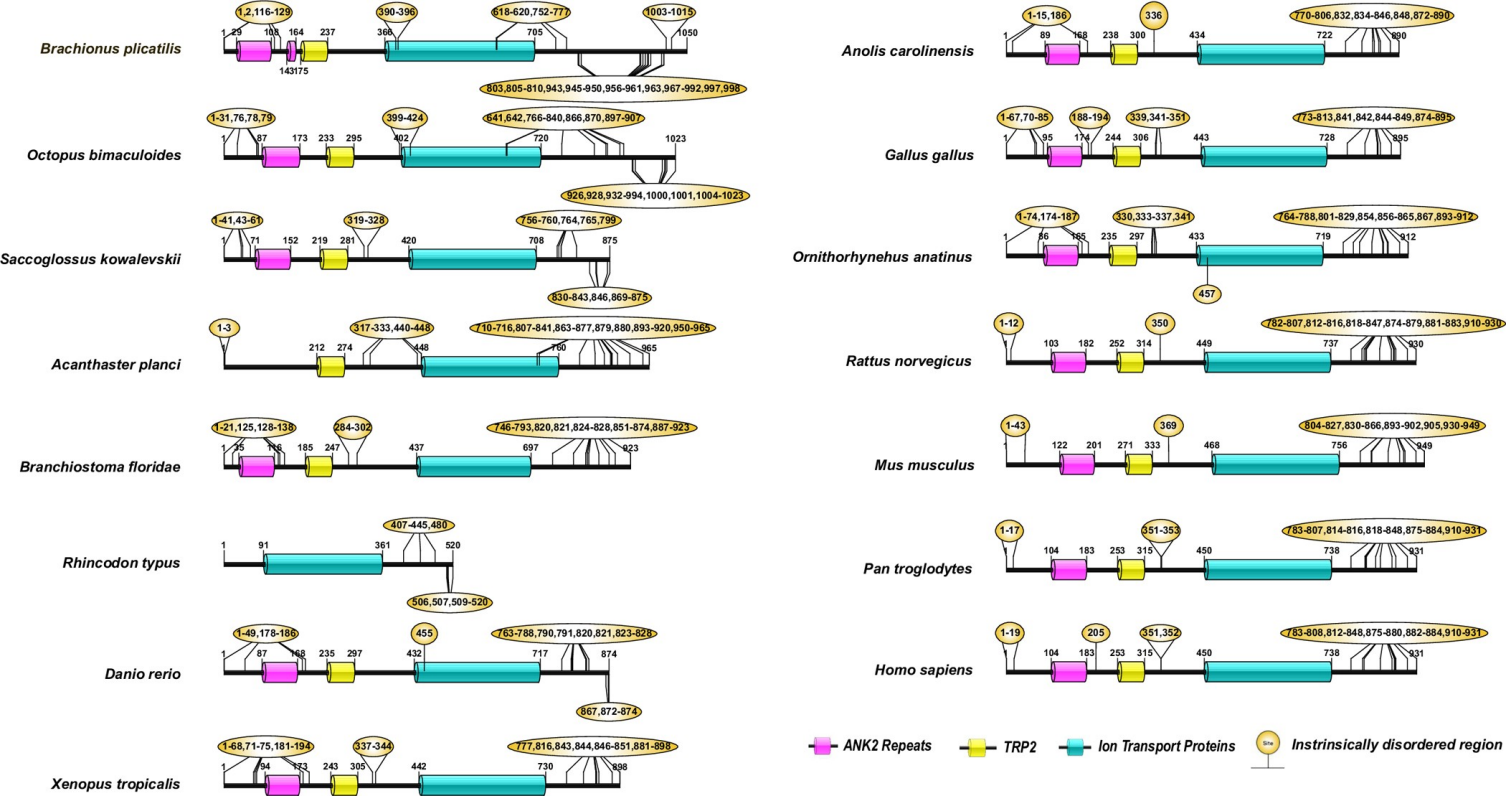
Domain organization in TRPC6, TRPC3, and TRPC7 sequences. The ankyrin repeats in the sequences are represented as a pink rectangle; similarly, the yellow rectangle represents TRP domains, and teal represents the ion transport domain, and the yellow bubbles represent the intrinsically disordered regions.

The human CD2AP consists of three Src Homology-3 (SH3) domains [19]. Pfam analysis of the CD2AP and the CINDR sequences revealed that these three SH3 domains are highly conserved (Fig 4). Human podocin consists of an SPFH domain (residues 127–299), while the rest of the sequence (residues 1–126 and 300–383) does not contain any known domains (Fig 5). Podocin from other vertebrates also possesses the SPFH domain, suggesting that it is highly conserved (Fig 6). The human TRPC6 sequence consists of three conserved ankyrin repeats in addition to a TRP domain and an ion transport domain [16]. Our analysis of the TRPC6 and its orthologs, including TRPC3 (Mollusca and Echinodermata) and TRPC7-like (Hemichordate), revealed that Ankyrin repeats, TRP, and ion transport domains were largely conserved (Fig 6). In the Chondrichthyes TRPC6, we observed only the ion transport domain but not ankyrin repeats or TRP domain. In Echinodermata, the TRPC3 protein is devoid of ankyrin repeats but possesses the ion transport and TRP domains (Fig 6).

### IURs are conserved motifs in SD proteins and their orthologs

SD proteins exist as large complexes via homo- and heterophilic interactions, and these interactions are predicted to be mediated by IURs [2,6,16]. Since the SD proteins and their orthologs share similar domains (Figs 2–6), we investigated whether IURs were conserved among them. DisProt consists of curated annotation for many proteins [20]. Nevertheless, in the current version of the DisProt, IURs information for the SD proteins and their orthologs is unavailable. Therefore, we used DISOPRED3.1 for predicting IURs in these sequences. Our results showed that most nephrin and orthologs have IURs at both N-and C-terminus and in a few Ig domains (Fig 2 and S1 and S2 Tables). However, in Rotifera, Mollusca, and Annelida, nephrin orthologs had IURs only at the C-terminus and in the Ig domains but not at the N-terminus (Fig 2). Nephrin sequence from amphibians had IURs even in the FN-3 domain. IURs are identified in the Ig3 and Ig5 domains and moderately in the CD80-like C2-set domains in the invertebrates. Interestingly in vertebrates, IURs were observed primarily on CD80-like C2-set domains.

IURs were noticed at both N- and C-terminus of KIRREL1 and its orthologs except in the Tardigrada, wherein IURs were observed only at C-terminus (Fig 3 and S1 and S2 Tables). Unlike nephrin, IURs were not observed in Ig domains of KIRREL1 except in Tardigrada, Mollusca, Annelida, Fishes, Reptilia, and *Pan troglodytes* (Mammals), wherein IURs were observed in only the Ig3 domain (Fig 3 and S1 and S2 Tables). In CD2AP and CINDR sequences, IURs constituted more than 50% of the sequence except for the regions that are a part of the first and third SH3 domains (Fig 4 and S1 and S2 Tables). Nevertheless, in the CD2AP sequence of Chondrichthyes, all the three SH3 domains were devoid of IURs, but the rest of the sequence had IURs. Podocin sequences from the Chondrichthyes to Mammalia showed IURs at both the N- and the C-terminuses (Fig 5 and S1 and S2 Tables). IURs in TRPC6 and its orthologs are predominantly localized to N- and C-terminuses with some intermittent regions excluding the ANK2, TRP2, and Ion transport domains. However, in the case of Rotifera, Mollusca, Fishes, and *Ornithorhynchus anatinus* (Mammals), IURs were observed in the ion transport protein domain. Further in Chondrichthyes, only C-terminal IURs were observed (Fig 6 and S1 and S2 Tables). Whereas IURs were restricted only to N- and the C-terminuses in TRPC6 from vertebrates (Amphibia-Mammalia). These results suggest that IURs are conserved motifs among the SD proteins and their orthologs.

## Discussion

Primitive nephron-like structures are identified in many invertebrate phyla suggesting an evolutionary connection among the vertebrate and invertebrate excretory units, which indicates that orthologous proteins similar to vertebrate SD proteins may be present in other metazoans. Therefore, in this study, we investigated the molecular evolution of human podocyte SD proteins, namely nephrin, KIRREL1, CD2AP, podocin, and TRPC6 proteins, that play a crucial role in aiding the SD integrity. Our analysis revealed that distributions of nephrin and KIRREL1 are diverse across several phyla in vertebrates and invertebrates, while CD2AP, podocin, and TRPC6 are confined mainly to vertebrates. Interestingly, SD proteins in vertebrates share significant sequence similarities but display subtle differences in the domain organization, especially in nephrin and KIRREL1. Furthermore, SD proteins and their orthologs share several conserved domains and IURs, indicating that the invertebrate orthologs could be the precursors of the vertebrate SD proteins.

In humans, nephrin is a 1241 amino acids transmembrane protein made up of eight Ig domains and an FN-3 domain [16]. In comparison, KIRREL is a 757 amino acids protein encoded by the *NEPH1* gene. In humans, KIRREL1 is a transmembrane protein consisting of an extracellular domain containing five IgG-like domains, a transmembrane domain, followed by a short intracellular domain [18]. Studies showed that Nephrin, along with KIRREL1, forms the characteristic zipper-like bridge between the adjacent foot process [7,18]. Although nephrin’s expression is observed in pancreatic islet cells and lymphoid tissues [21–23], KIRREL1 expression occurs exclusively in the kidneys [24]. Mutations or knockdown of the gene encoding the nephrin caused Finnish-type congenital nephrotic syndrome and improper development of coronary arteries in human and mice embryos [17,24,25].

In contrast, mice lacking the *NEPH1* gene developed prenatal lethality and proteinuria [24]. Although nephrin and KIRREL1 distribution in metazoans are diverse, their function in invertebrates is poorly understood. Earlier studies showed that Aves does not have a gene to express nephrin but instead adherens junction proteins, namely N- cadherin and α- & β-catenins substitute for the function of nephrin [26,27]. Further, it is also speculated that the KIRREL family of proteins may also substitute for nephrin’s role in Aves [28]. Therefore, it is not surprising that our studies could not identify any orthologs for nephrin in Aves. CD80-like C2-set domains are more in vertebrate nephrin sequences compared with invertebrates [13,29,30]. Due to the increased number of CD80-like C2-set Ig domains and the IURs distribution in them, we assume that nephrin in vertebrates may facilitate stronger homophilic interactions between neighboring nephrin/KIRREL1 molecules. However, we are unsure how Ig domains and IURs in KIRREL1 may partake in these interactions [31,32]. Although we found an evolutionary relationship between vertebrate nephrin and KIRREL1 sequences based on the phylogenetic analysis, the bootstrapping values are not entirely reliable, particularly when inferring phylogenetic relationships within invertebrates.

Human CD2AP is a 639 residues protein primarily identified as an actin-binding cytoplasmic ligand for CD2 in T-cells and natural killer cells [33–35]. CD2AP acts as an acting binding adaptor protein and helps nephrin/KIRREL1 signaling in podocytes [36]. CD2AP is essential for SD integrity and podocyte permselectivity since CD2AP knockout caused mice to develop nephrotic syndrome [37]. Although CD2AP shares ~50% similarity with CIN85, which belongs to SH3 domain-containing kinase-binding protein 1 (SH3KBP1), our analysis identified CINDR as the only CD2AP ortholog in Arthropoda. The adaptor molecules characteristically consist of three SH3 domains, a proline-rich motif, and a coiled-coil region [19,38,39].

Positional cloning identified *NPHS2* encodes podocin which is 383 residues integral membrane [9]. Podocin acts as a scaffolding molecule and provides structural integrity to the SD by forming a macromolecular complex with nephrin, CD2AP, TRPC6, and KIRREL1 [10,40]. Like nephrin and KIRREL1, podocin associates both as homo and heteromeric complex, like its family members [6,41]. Members of the stomatin family are observed in primitive (bacteria) and complex organisms (metazoans). Nevertheless, our study could not identify podocin orthologs in invertebrates despite podocin’s significant homology (~40%) with the other stomatin family proteins through its SPFH domain”.

The human TRPC6 is a 931 residues cation transport channel associated with smooth muscle contraction, pulmonary endothelial permeability, neuronal protection against ischemia, and podocytes’ structure and function [42]. TRPC6 and its related TRPC channels are a part of a more prominent family of TRP proteins involved majorly in chemo- and mechanosensation [43,44]. Based on the sequence similarity, the TRPC6, TRPC3, and TRPC7 proteins share appreciable homology. Furthermore, the TRPC proteins share several conserved regions, namely; a) Ankyrin (ANK) repeats, b) coiled-coil domain, c) a 25 residues TRP domain, d) proline-rich sequence, followed by e) a calmodulin and IP3 receptor-binding region (CIRB region), and f) C-terminal coiled-coiled domain [44]. Therefore, it is predictable that the reciprocal best-hit method retrieved TRPC3 and TRPC7 proteins as the orthologs of TRPC6 and that these sequences share multiple conserved domains [45,46].

IURs are areas in the protein sequences that do not adopt any secondary structure conformation in isolation. However, in the presence of an interacting partner or a suitable ligand, IURs adopt ordered structures [47]. Further, IURs are known to mediate protein-protein interactions and signaling events [48]. It is suggested that SD proteins consist of IURs through which they associate into large complexes. Since SD proteins are predicted to have IURs, we were interested to know if IURs are conserved in the SD orthologs. Our results have shown that IURs are also conserved across the SD orthologs, further ascertaining the evolutionary link between SD proteins and their orthologs. The interaction sites determined from the co-immunoprecipitation studies and the strategic location of IURs in the SD proteins make us assume that IURs may promote homomeric and heteromeric interactions [6,10,16,30,36,41,44,49,50].

In summary, this study provides novel insights between the vertebrate SD proteins and the invertebrate orthologs. We show that the unique domains and IURs present in the SD proteins are highly conserved. We speculate that the orthologs sequences identified in the invertebrate phyla may be the precursors for the vertebrate SD proteins. The limitation of our study is that the reliability of bootstrap statistical validation that we performed for deducing the phylogenetic relationships in invertebrates may not be reliable due to limited genome availability in metazoans.

## Supporting information

S1 Table Amino acid residues predicted as intrinsically unstructured regions (IURs) and IUR-binding domain (BD) by the DISOPRED 3.1 tool of the PSI-PRED server the slit-diaphragm proteins and their orthologs in various metazoans. (DOCX)

S2 Table DISOPRED 3.1 prediction confidence for each residue in Nephrin, KIRREL1, CD2AP, podocin, and TRPC6 and their orthologs sequences. A predicted threshold value of ≥0.5 for residue indicates it be intrinsically unstructured.
(XLSX)
